# Safety of Electronic Cigarette Use During Breastfeeding: Qualitative Study Using Online Forum Discussions

**DOI:** 10.2196/11506

**Published:** 2019-08-12

**Authors:** Emily Jade Johnston, Katarzyna Campbell, Tim Coleman, Sarah Lewis, Sophie Orton, Sue Cooper

**Affiliations:** 1 Division of Primary Care School of Medicine University of Nottingham Nottingham United Kingdom; 2 Division of Epidemiology and Public Health School of Medicine University of Nottingham Nottingham United Kingdom

**Keywords:** e-cigarette, online forum, postpartum relapse, smoking, breastfeeding, forum data

## Abstract

**Background:**

Electronic cigarettes (e-cigs) are an increasingly popular alternative to smoking, helping to prevent relapse in those trying to quit and with the potential to reduce harm as they are likely to be safer than standard cigarettes. Many women return to smoking in the postpartum period having stopped during pregnancy, and while this can affect their decisions about breastfeeding, little is known about women’s opinions on using e-cigs during this period.

**Objective:**

The aim of this study is to explore online forum users’ current attitudes, motivations, and barriers to postpartum e-cig use, particularly as a breastfeeding mother.

**Methods:**

Data were collected via publicly accessible (identified by Google search) online forum discussions, and *a priori* codes identified. All transcripts were entered into NVivo for analysis, with a template approach to thematic analysis being used to code all transcripts from which themes were derived.

**Results:**

Four themes were identified: use, perceived risk, social support and evidence, with a number of subthemes identified within these. Women were using e-cigs to prevent postpartum return to smoking, but opinions on their safety were conflicting. They were concerned about possible transfer of harmful products from e-cigs via breastmilk and secondhand exposure, so they were actively seeking and sharing information on e-cigs from a variety of sources. Although some women were supportive of e-cig use, others provided harsh judgement for mothers who used them.

**Conclusions:**

E-cigs have the potential to reduce the number of women who return to smoking in the postpartum period and potentially improve breastfeeding rates, if breastfeeding mothers have access to relevant and reliable information. Health care providers should consider discussing e-cigs with mothers at risk of returning to smoking in the postpartum period.

## Introduction

Maternal smoking and low breastfeeding rates are both major public health concerns relating to the postpartum period, with health implications for both the mother and her child [1-8]. While many mothers are able to quit smoking during pregnancy, a substantial proportion will return to smoking by six months postpartum [9]. The latest UK statistics show that, despite 81% of mothers initiating breastfeeding at birth, by six months only 1% of UK infants are still breastfed [10].

Studies have consistently reported associations between smoking behavior (abstinence) and breastfeeding patterns [11-15], with the intention to breastfeed acting as a precipitating factor for reducing postpartum return to smoking, and then the initiation and continuation of breastfeeding being positively associated with smoking abstinence postpartum [16,17]. The intention to return to smoking is one of the strongest predictors of the intention not to breastfeed and the early cessation (<3 months postpartum) of breastfeeding [13,18]. These associations may be partly explained by confounders like sociodemographic factors [19,20], but they may also be attributable to concerns regarding safety of smoking while breastfeeding. This is despite online information from both the American Pediatrics Association [21,22] and the National Health Service (NHS) [23] that promotes the continuation of breastfeeding even if the mother smokes.

A relatively new product that may be useful for preventing return to smoking or supporting cessation of smoking is the electronic cigarette (e-cig) [24]. E-cigs are handheld devices that produce an aerosolized mixture from a solution typically containing concentrated nicotine, flavoring chemicals and propylene glycol [25]. The user draws a deep breath and inhales the vaporized liquid (a process known as vaping), which creates a similar experience to smoking combustible tobacco [26]. Although we accept that the long-term health effects of e-cigs cannot be fully assessed given the relatively short time they have been available for use, the current research suggests that e-cigs are likely safer than smoking traditional cigarettes and their use is proposed as a harm reduction tool for smokers [27,28].

Online forums are a popular and accessible community for mothers to discuss, debate and share opinions on anything relating to motherhood [29]. A method of research known as infodemiology analyzes online discussions to inform public health research [30,31], and previous studies of this type have studied e-cigs [32,33], breastfeeding [34] and parental health-seeking behavior [35]. With the popularity of parenting forums and increased use of social media for health-seeking advice, this study analyzed parenting forum discussions to determine opinions on e-cig use.

Very little research thus far has examined the use of e-cigs amongst postpartum women, with the current research available focusing on the use of e-cigs in pregnancy. Therefore, we aimed to explore online forum users’ current attitudes, motivators and barriers to using e-cigs as a breastfeeding mother through the analysis of data extracted from online parenting forums.

## Methods

### Overview

This was an infodemiological study [30] using online forum data. Qualitative analysis of discussions on online parenting forums has previously been used to explore a wide range of context-specific behaviors, attitudes and beliefs [36,37], including the use of e-cigs during pregnancy [38]. Online support groups provide specific benefits of a virtual group membership compared to a physical group membership, such as being accessible 24-hours a day, 7 days a week, being free to join and participate in, lacking geographical barriers and offering anonymity [39-43]. The use of online support groups or forums can have potentially empowering effects on those who use them by, for example, providing health information, information sharing and input for individuals to make health-based decisions [39]. Online forums can also offer a safe place to discuss sensitive topics or topics in which a person feels they may be judged [44] and have been used for discussions regarding smoking [45]. Therefore, the use of parenting forums is a valuable source of data on what women think about health-related risks and their health-related decision making [46-48].

### Inclusion Criteria

Due to varying guidelines between countries on e-cig use, only UK-based forums were used as the United Kingdom already has guidance recommending the use of e-cigs rather than traditional cigarettes during pregnancy [28]. The eligibility for inclusion of a thread (a continuous discussion on a forum) in the final analysis were: (1) it was posted to a forum that is open to public use, without the need to sign up or log in to read posts; (2) it was posted to parenting forums not affiliated with vaping or tobacco companies; (3) it contained a minimum of four unique contributors to the discussion; and (4) the discussion needed to include the mention of either e-cig use or vaping as well as breastfeeding.

### Search Strategy

The key words for e-cigs (Textbox 1) were combined using the operator *AND* with key words for breastfeeding, and then searched using the search operator (a Google-based command to filter results) *site: sampleforum.co.uk* via Google search engine.

**Key words (search terms).**

Key words: electronic cigarette

- E-Cig(s)
- E-cig(s)
- Electronic cigarette(s)
- Vaping
- Vape(s)

AND

Key words: breastfeeding

- Breastfeeding
- BF(ing)
- Nurse/Nursing
- Breastmilk
- Feeding

The use of search operators was the most effective and thorough way to ensure relevant discussions were obtained while ignoring forums owned by specific groups who may have had competing interests, such as e-cig manufacturers or tobacco companies. These sites were identified by screening the URL name and home page.

A total of 597 Google results were returned using the above search terms, and searches were then adapted to exclude *pregnancy* and *trying to conceive*, in line with the aims of the research. The threads used in analysis were then transcribed for NVivo11.

In the analysis, abbreviations within quotes were expanded in squared brackets, and the data source was identified by the thread (T) and the numbered data set.

### Ethical Considerations and Data Collection

Informed individual consent was not obtained as the data were publicly posted on a large forum [49-52]. The British Psychological Society [53] recognizes that although informed consent might not be achievable in this context, certain steps can be taken to protect the participants. Therefore, only data from publicly accessible forums, where users are made aware during the initial sign-up process that all posts are open to public access, were used [54]. Furthermore, as comments on large public forums are less identifiable than those on smaller, private online communities [55], data was only obtained from large forums (for the purposes of this research, a large forum was defined as forums with over 1000 members) [55]. All contributing users were randomly assigned a new name to protect their identity, then names of people, places and institutions were removed from quotes and finally quotations were corrected for spelling and kept brief to reduce the possibility of them being traced back to the original poster.

Ethical approval was obtained from the University of Nottingham Medical School Research Ethics Committee.

### Analysis

Template analysis (a template approach to thematic analysis), following the guidelines outlined by King [51,56], was used to analyze the data. Due to the use of *a priori* codes, this permits the analysis of the textual data that had been produced for “a different purpose in a different context” [57]. When analyzing large online support group datasets, template analysis is useful for comparing the perspectives of different contributors. In the current study, the initial template of *a priori* codes was used to code each transcript with codes being continually modified or expanded. After the last transcript was coded, a final version of the template was used to recode all transcripts (Multimedia Appendix 1). A mind map was also created to show integrative relationships and prevalence (Multimedia Appendix 2).

## Results

### Overview

Of the eight parenting forums identified, two met the inclusion criteria. Using the search operator *site: sampleforum.co.uk* for the two forums, a total of 95 discussion threads were identified and screened for inclusion. From those threads, 39 of them were duplicate results and 46 did not meet the inclusion criteria, leaving a total of 10 results to be analyzed (Table 1).

**Table 1:** Threads selected for analysis.

Thread number	Opening post title	Website	Sub-group heading	Comments
T^a^1	Vaping whilst Breast feeding?	Babycentre	Vapers Lounge	13
T2	Ecigarette and breastfeeding :(	Babycentre	June 2016 Birth Club	6
T3	Smoking while breastfeeding?	Babycentre	September 2015 Birth Club	19
T4	Does anyone vape?	Babycentre	October 2015 Birth Club	10
T5	Today I am..	Babycentre	February 2015 Birth Club	48
T6	(AIBU^b^?) To smoke an electronic cigarette whilst breastfeeding?	Mumsnet	Am I Being Unreasonable? (AIBU)	23
T7	(AIBU) To use the vape? For friend?	Mumsnet	Am I Being Unreasonable? (AIBU)	6
T8	(AIBU) To use electronic cigarettes even though I'm BF^c^?	Mumsnet	Am I Being Unreasonable? (AIBU)	55
T9	(AIBU) To ask DH^d^ to stop vaping?	Mumsnet	Am I Being Unreasonable? (AIBU)	129
T10	(AIBU) To not give up smoking just yet?	Mumsnet	Am I Being Unreasonable? (AIBU)	39

^a^T: thread.

^b^AIBU: am I being unreasonable.

^c^BF: breastfeeding.

^d^DH: dear husband.

Four main themes were identified within the transcripts: use, perceived risk, social support, and evidence, with each of these having a number of subthemes.

### Use

First, three subthemes were identified for the main theme of use, which included preventing returning to smoking, quitting smoking and motivation for use.

Based on the results, women were using e-cigs postpartum in a variety of ways. Some reported using them to prevent returning to smoking and described having cravings postpartum that were often associated with specific triggers such as the demands of motherhood, mental health issues or relationship problems. Motivation for use was a separate subtheme, as this applied to those who had already returned to smoking postpartum as well as those who were still abstinent, with women identifying e-cigs as preferable to smoking. Some women reported quitting suddenly and completely throughout pregnancy, but then they experienced cravings postpartum and found these could be alleviated by e-cig use:

Before pregnancy I used to smoke roll ups but quit when I found out I was pregnant! But after giving birth I started craving badly so decided that rather than smoking again I would try e-cig.

Cressida, T2

Some women had used an e-cig to quit during pregnancy, but then continued use of the e-cig postpartum had prevented them from returning to smoking. Others, however, did not manage to achieve abstinence during pregnancy or had already returned to smoking postpartum, while some had planned to return to smoking postpartum as they enjoyed it. The following quote highlights the experience of one woman who had already identified that she enjoyed smoking and didn’t want to lose that experience, but had found an e-cig to be a suitable alternative:

I didn't want to quit, I liked smoking. Bought an e-cig and did 24hrs on it and thought well I can't go back to smoking now. That was 9 months ago and I haven't smoked at all.

Sakina, T10

As identified above, women were able to identify what motivated them to seek out an alternative method of nicotine delivery. These reasons were mainly related to the context-specific issues attributed to new motherhood, such as lack of sleep, stress, loss of identity and relationship difficulties, as discussed below:

She is going through a massively stressful time right now and struggling to cope. She borrowed her mum's vape and loved it, felt totally better and less stressed straight away.

Leah, T5

### Perceived Risk and Strategies to Mitigate Risk

The theme of perceived risk had four subthemes identified, including behavioral strategies, psychological strategies, physiological effects and environmental risks.

Although women were using e-cigs postpartum they still had concerns, with the perceived risks of vaping often compared to the risks of smoking:

They are not unregulated, we know what's in them and they are at least 95% safer than tobacco.

Talitha, T6

Sometimes the e-cigs were compared favorably to regular cigarettes and information on them was used to make assumptions on the safety of e-cigs, such as the guidance on smoking and breastfeeding being used to argue the safety of vaping and breastfeeding:

They say it's better for a smoker to smoke and breastfeed than not to breastfeed at all, so I should think the same applies to e-cigs.

Delilah, T4

However, there were also unfavorable comparisons, such as the health detriments of smoking being projected onto vaping:

I'm not an anxious or risk averse person, really. It's just the link between smoking and SIDS [sudden infant death syndrome] is so strong. What if you do continue to breathe out something for hours after vaping? If in twenty years they turn round and say vaping and co-sleeping causes x, and our baby had x?

Acacia, T7

Many of these comparisons were related to nicotine content in e-cigs, with one forum user advising another that it was safe for her husband to vape as he was not the one breastfeeding:

The most harmful thing in e cigarettes is the nicotine. Unless your DH [dear husband] plans on doing the breastfeeding, it isnt going to harm your baby.

Daffodil, T7

There were also perceived risks associated with the physiological effect of vaping on breastmilk, with discussions on what was likely to transfer to the infant if the mother vaped. There were comments about unknown substances that may be harmful if transferred to an infant; however, the most commonly discussed concerns were about nicotine and the perceived health risks associated with passing nicotine to the baby. The concern was often mixed with judgement, with the emphasis being that a good mother would not smoke or vape:

You're basically asking, “AIBU [Am I Being Unreasonable] to feed my baby small amounts of nicotine”? What do you think OP [opening poster]?

Tirzah , T6

Mothers were also informed their infant would develop an addiction to nicotine, in that the infant would *‘feel like they want a fag [cigarette]’* and would suffer *‘withdrawal’* from nicotine when breastfeeding ceased.

The concept of risk came with a variety of strategies to manage these perceived risks. Behavioral strategies were one example, and they involved altering behaviors to reduce exposure to vapor for infants. Alterations included only vaping outdoors or in a separate room, choosing low nicotine juice, or timing vaping around the infant’s feeds to allow the maximum time to pass between vaping and the infant receiving inhaled components via breastmilk.

Psychological strategies were also used, which involved justifying any perceived risk in a way that presented a woman’s choice to vape in a more favorable light, such as explaining that without vaping they would be stressed, which would be worse for their baby. They also justified the perceived risks of vaping by comparing it to more accepted health behaviors such as drinking coffee, as illustrated in the following quote:

Nicotine is in the same drug classification as caffeine so its only as bad as anyone that drinks coffee and breastfeeds.

Helena, T2

As well as specific risks to infants via the breast milk, there were wider concerns for risks from the environmental exposure to vapor. This was mainly founded on the basis of the harm from passive smoking, with women more concerned about the exposure to secondhand vapor based on the known harm from secondhand smoke.

### Social Support

The third main theme had three subthemes identified within it, including informational, emotional, and instrumental social support.

While discussing risk on the forums, women were also seeking and giving support to one another about vaping, with the social support they received varying in nature. In many ways, the forum users offered positive social support to women who were vaping or considering an e-cig. There was also informational support, such as giving advice on which products to use or how best to use an e-cig. Informational support was often guided by the woman’s own experience of vaping and included positive messages to support women, especially those who were trying to quit smoking.

Emotional support came from supportive comments about posters’ own experiences as well as the experiences of others who had quit smoking and the health benefits they experienced, or by reassuring a vaper that they would not be judged for vaping. The following forum user discussed her partner’s experience of vaping and how she viewed it positively:

I would much rather see him vape than smoke and he no longer wheezes when lying down, he is much fitter and it's the first time he has gone longer than a week without smoking. I think he is coming onto three years now.

Bryony, T7

However, not all posts were positive and supportive. There were instances of harsh judgement of vaping mothers, or indeed a mother’s harsh judgement of herself. There were accusations of not putting their infant’s needs before their own and the insinuation that by vaping they were somehow encouraging their child to learn unhealthy coping techniques:

Why would anyone condone it? Very strange. She is teaching her son an unhealthy way of coping with normal life stress.

Tabitha, T6

The varying forms of support often led to a polarized divide among forum users, with strong views expressed among both those who were provaping and antivaping.

Instrumental support was also identified, which included directing women to the best places to buy products or other forums to use for more information. This was also evident from those opposed to smoking, as they would direct women to alternative products or behaviors to remain smoke-free, including traditional nicotine replacement theory (NRT) use, self-help materials or sometimes more comical ways of both parents remaining smoke free:

Reward him with...I dunno. Doughnuts or something. I'd suggest BJs [oral sex] but then I remember how pregnant you are.

Xanthe, T7

### Evidence

Finally, five subthemes were identified for the main theme of evidence, which included professional, non-professional, anecdotal, lack of evidence and mistrust or uncertainty of evidence. This theme showed that women accessed a wide variety of sources of information to inform their arguments and opinions, and then interpreted and communicated their understanding of this evidence on forums (sometimes inaccurately).

Professional evidence came from academic articles or via professional websites such as the National Health Service (NHS) and Public Health England (PHE). This evidence was often misinterpreted, particularly by those who were opposed to vaping. One example was an article available on the NHS website about popcorn lung that was incorrectly cited several times across transcripts as evidence of e-cigs being harmful. A further example of using professional evidence is the following poster, who linked a paper by Farsalinos and Polosa [58]:

If you want decent info on the risks and benefits of vaping this is a good place to start. You can access the whole paper for free if you create an account.

Jael, T4

The most frequently quoted evidence was from nonprofessional sources. This included media articles such as blog posts and newspaper articles, but also links to social media profiles and discussions. There were also examples of websites, like Wikipedia, being cited as sources of evidence against vaping, which was met with ridicule by some provape forum users. The nonprofessional evidence was mostly quoted by those opposed to vaping, whereas professional evidence was equally shared by those both for and against vaping.

Anecdotal evidence was also shared by both sides and appeared to be the most substantial form of evidence accepted by women. The women were often more responsive to the experiences and stories from other forum users than they were to other forms of evidence available, and these forms of evidence often appeared to be more persuasive:

Anecdotally I can tell you that when my ExH [Ex-husband] vaped, our cats fled from the vapour and I hated the idea of him vaping inside near the cats. I'd be even more concerned about a baby.

Grace, T4

I do, I feel so much better too, no coughs or colds. I am positive that e cigs are much much less dangerous than cigarettes and think maybe you're being a bit over anxious.

Jonquil, T7

As well as sharing, quoting and interpreting evidence, there was also a general discussion on the lack of evidence available about the safety of e-cigs. This was most often attributed to a lack of empirical evidence of the long-term effects of vaping. Women were anxious to read information that related specifically to their situation and talked about lack of evidence on vaping and breastfeeding or vaping around young children.

This lack of evidence specific to new mothers was also displayed in the final subtheme of mistrust and uncertainty. In the following quote a forum user highlights the use of thalidomide in pregnancy, and how this was perceived as safe:

95% safer, Not 100% safe then? Not that long ago the NHS also said Thalidomide was safe. Look how that ended.

Camelia, T7

This is evidence of women looking for evidence that relates to their specific circumstances by using the comparison of a professional recommendation that resulted in infant harm. It wasn’t just mistrust at the science itself, but also the institutions that make the recommendations:

And PHE [Public Health England] have been criticised for their supportive stance on e-cigs. They are very keen to get tobacco smoking down so I can see why they would be supportive.

Joy, T6

## Discussion

An infodemiologiocal approach was taken for this research, which is the analysis of data on internet forum sites for public health research [30,31]. This research is the first to describe how women are accessing information about e-cig use during the postpartum period and is the first to provide evidence of women proactively using e-cigs to prevent returning to cigarette smoking and to aid smoking cessation, particularly as breastfeeding mothers. Women have concerns regarding the potential risks of using an e-cig and utilize online forums to discuss these risks with other women. This type of forum provides both positive and negative social support.

The themes show that women are accessing both lay and professional information on e-cig safety and their use via multiple sources, but this information is not necessarily being interpreted correctly, or it is being met with a degree of mistrust and uncertainty. There are conflicting opinions on the use of e-cigs while breastfeeding, mainly due to health concerns regarding what may be transferred from e-cig to the breastmilk, and then to the infant, as well as concerns about harm from secondhand vapor exposure.

There are limitations to this form of infodemiological research, with the use of online forum data forgoing the possibility of following up with individual users or seeking clarification on the meaning of their words, which increases the risk of bias during coding. It is also impossible to establish the validity of posts, like being completely confident that a user who identifies as a breastfeeding mother is, in fact, a breastfeeding mother. The transferability of these themes to the general postpartum population is limited due to both the exclusive participation of forum users and also that all the transcripts came from only two parenting forums. There is also no way to completely establish the authenticity of the users on the forum or whether they have connections within the e-cig or tobacco industry. On the other hand, the use of online data has several strengths, like that discussions are free from the response bias that may be present within interviews, and that forum data provides discourse that has been written with the intention of expressing and debating opinion for the purposes of discussion rather than research. The use of forum data provides in-depth qualitative data, and in other research, the use of a discussion analysis tool found that online interactions involving conflicting viewpoints promoted more discussion and critical thinking [59]. Our research is novel in both subject matter and approach, and therefore should be treated as an exploratory qualitative piece upon which further research can be built. Thus far, this is the only piece of work that considers the motivators, barriers and opinions of breastfeeding mothers using e-cigs postpartum.

This work has improved our understanding of how and why women use e-cigs in the postpartum. For the first time we are able to understand and explore what evidence women are accessing to inform themselves about e-cigs and how this information is then interpreted. It also provides the first evidence of women perceiving their use of e-cigs postpartum to be preventative of a return to smoking. We are also better able to understand the concerns women have around the impact of e-cigs on infant health, in particular that a misattribution of nicotine as the most harmful substance transferred to infants via a smoking mother’s milk is sometimes an obstacle to the use of e-cigs postpartum.

Previous research has highlighted that some women perceive that smoking affects the quality of their breastmilk in a way that is detrimental to their infant’s health [15,60] despite the previous decade of recommendations from professional health bodies encouraging women who do smoke to continue breastfeeding [23]. This current research helps us better understand the fears women have in relation to e-cig use, most notably in relation to the perceived lack of consistent, evidence-based information about e-cig safety and the effects of nicotine transferring through breastmilk. By addressing these concerns, we could improve the acceptability of using a vape alternative for women who are breastfeeding and also smoke, while minimizing harm to the mother and infant and reducing their fears regarding their child’s exposure to vape constituents via breastmilk.

Prior work using forum data considering e-cig use during pregnancy [31] identified three distinct themes explaining the ways in which forum users debated the use of e-cigs while pregnant: (1) quitting (nicotine) abruptly and completely is unsafe; (2) vaping is the lesser of two evils; and (3) vaping is not worth the risk. The authors concluded that women perceived their addiction to cigarettes as more than just a nicotine addiction, and that the behavioral aspects of smoking were also important, hence the potential for e-cigs. In our forum transcripts, women reported using e-cigs either to prevent returning to smoking or to quit smoking after having already relapsed. Returning to smoking, or perceived likelihood of returning to smoking, was often triggered by the demands of motherhood, perceived stress or the feeling of needing some personal time. It is interesting that some women are choosing an e-cig for similar reasons that have previously been identified for why women return to smoking traditional cigarettes, including smoking for relief and nostalgia for their former self [61]. Although evidence is limited, it is suggestive that women who use e-cigs during pregnancy are still likely to return to cigarette smoking, with one qualitative study partly attributing this to a lack of professional consensus within healthcare on the safety of e-cigs [62]. Lack of consistent and transparent information from professional health sources is a significant barrier to e-cig use postpartum, an issue that needs to be addressed given the success some women have reported on using e-cigs to prevent a return to smoking.

In our study, women also displayed mixed views on e-cig safety. The majority of users accepted that e-cigs were probably safer than cigarettes; however, there was a lot of skepticism and mistrust of the evidence for this. Health bodies such as PHE and the NHS were classed as biased due to their targets of reducing cigarette smoking, and comparisons were made regarding previous health recommendations that have since proven to be detrimental. Women were accessing scientific journals to learn more about e-cigs; however, it was often mistranslated. News media stories are often shared among online groups if a headline is particularly provocative, and even when these stories were discredited users felt that these fears must be based on something. Lack of evidence or mistrust of the current evidence appears to be a barrier for the use of e-cigs as a harm reduction tool during the postpartum period, which often gives rise to the thought process that it is better to deal with the familiar than risk the unknown. This skepticism is not confined to e-cigs, as previous research has identified that some women believe NRT patches to be harmful and that smoking is preferable to using them [63].

Uncertainty regarding e-cig safety often led to women discussing the concept of risk either in terms of comparison to smoking or in justifying the perceived risk. This individual assessment of risk is not unique to e-cig use and is attributed to a knowledge deficit between professionals and the lay public [64]. The risk assessment formed by lay people is complex, situationally influenced and reflects their personal values [65], and all of this is particularly relevant when considering the morality of motherhood and the negative attitudes some women hold towards vaping while breastfeeding. For example, the negative attitude towards government-backed advice on risk is assumed to be due to perceived exclusion from science-led and political decision-making [66]. With this in mind, involving women in discussions about e-cig use and safety within usual postnatal visits could help them make an informed choice on e-cig use.

This knowledge deficit could explain the reliance on unverified evidence from social media, news publications or web content found within this study. These sources of information are written to inform a general population but are also written to be read with ease, so this may be why women are engaging more with this type of evidence. There is also a reliance on others for information, with women seeking support and advice from health care professionals but also from other mothers. It is unsurprising that new mothers would seek information that is easy to access and easy to read, but the use of online forums also provides anonymity, which provides some form of protection of self while receiving or giving information [67]. Therefore, despite judgement from other mums, the ability to remain anonymous allows a mother to still have some perceived control over how those around her perceive her morally and ethically.

The concept of risk is a subjective one, and while there was much discussion of it, this risk was not defined apart from discussions on nicotine. There were suggestions of harm drawn from media conclusions, but risk itself was often discussed as a general term. Other lifestyle behaviors such as alcohol and caffeine consumption were often used as a comparison to justify this risk concept, with forum users suggesting that if these behaviors were acceptable for mothers then vaping was also acceptable. However, the risks of nicotine exposure to the baby were one of the defined examples of risk. There were unsubstantiated attributions that nicotine caused SIDS, fears of infants becoming addicted to nicotine and being forced to experience withdrawal once breastfeeding ceased, and concerns that using e-cigs to manage the mother’s mental health needs (such as stress) would lead to children who grew up exposed to unhealthy coping mechanisms. The exposure of infants to nicotine was also the subject of judgement. Some women would argue that a mother asking if it was acceptable to vape was actually asking if it was acceptable to feed her baby nicotine, suggesting that the nicotine became a deliberate exposure rather than a byproduct of the breastmilk. This concern regarding nicotine acted as a barrier to the use of e-cigs postpartum, and although some women acknowledged the known harmful substances in cigarettes, their primary concern seemed to be nicotine. Harmful effects from nicotine are not fully understood but are likely to be minimal compared to the effect of other compounds. Although it is accepted as an extremely addictive substance [68], there are far more worrying compounds within cigarette smoke, which research suggests are either not present in e-cigs or are present at significantly lower levels [69]. There is still limited empirical data on the safety and composition of e-cig vapor; however, there has been some promising toxicity testing that has evaluated the chemical nature of the vapor generated from e–cigarettes [69]. Despite the identification of certain toxicants within e-cig vapor, these levels are <1% of the levels present in cigarette smoke. E-cigs therefore have potential as a harm reduction tool, as confirmed by the PHE report [70].

This research closely relates to the “good mother” social construct [71], as shown with the various justifications of perceived risk, or the moralized stances against the use of any nicotine-containing products by a breastfeeding mother. The role of the mother is one that is subject to historical and cultural experiences, and social networks provide a framework to help make sense of culturally defined experiences and responsibilities [72]. The use of an online forum varies slightly from this by bringing together women from various socioeconomic backgrounds, ages, experiences and cultures to discuss breastfeeding. Therefore, the social constructs of a “good mother” are more explicit, particularly for infant feeding [73], whereby a “good mother” is synonymous with a breastfeeding mother without considering any cultural or environmental context [71]. The justification of risk here is similar to that of mothers who justify smoking by claiming that it is for their baby’s sake [74], that is, it is better for the baby to have a mother who isn’t stressed or is more alert.

In conclusion, this study has shown women hold a mixture of views on the acceptability of vaping as a mother, but some women are using (or are interested in using) e-cigs in the postpartum period. They are seeking, and need, more reliable information to facilitate their use, especially when breastfeeding. Therefore, we need further research that considers how women could have opportunities to ask and receive advice, perhaps by opening a dialogue on e-cigs between mothers and health care providers which could potentially reduce rates of maternal smoking and increase breastfeeding rates.

## Appendix

Multimedia Appendix 1 Final coding template.

Multimedia Appendix 2 Mind Map. Integrative themes - mapping.

## Abbreviations

AIBU: am I being unreasonable?
DH: dear husband
e-cig: electronic cigarette
ExH: ex-husband
PHE: Public Health England
NHS: National Health Service
NIHR SPCR: National Institute for Health Research School for Primary Care Research
NRT: nicotine replacement theory
SIDS: sudden infant death syndrome
T: thread
