# Supplementary material for: Safety of Electronic Cigarette Use During Breastfeeding: Qualitative Study Using Online Forum Discussions
Source: J Med Internet Res. 2019 Aug 12;21(8):e11506. doi: 10.2196/11506 (PMC6709563; doi:10.2196/11506)

Appendix 1 – Final coding template

| 1st level | | 2nd level | |
|---|---|---|---|
| Evidence | The sharing of evidence from various sources to support the argument for the use of E-cigarettes or the dis-use of them for breastfeeding mothers. | Anecdotal | Evidence is presented from personal opinions, or through the sharing of non-scientific articles. May include online sources which are not government certified authorities on health, or media stories |
| | | Professional | Evidence is presented from scientific sources or from health professionals. It may be that the health professional quotes do not support the scientific knowledge, but will still be reflected here as an authority on health. Online sources such as NHS based sites will be included here, as well as academic citations. |
| | | Lack of evidence | Asserting that conclusions cannot be drawn due to the lack of relevant evidence. This may include accepting recent evidence, but wanting long term evidence, or not accepting enough evidence to make an informed choice. |
| Social Support | Includes areas of support such as online groups, messages of support and the sharing of personal experiences of both smoking, quitting, remaining smoke free and relapse | Advice: seeking and giving | Asking for advice and recommendations as well as giving advice and recommendations. Unlike the evidence category this is based on personal opinion, experience or recommendation from others. |
| | | Validation to us or not use | The behaviours and opinions of others validate a woman's choice to vape and breastfeed or choice not to. May cause divisions in the posters. |
| | | Judgement | May be of self or of others – includes feelings of shame, embarrassment or concerns about the ability to be a good mother. May also include comments designed to shame mothers who vape or project own parenting ideals on to others regarding vaping and breastfeeding. |
| Breast Milk & transference | Discussions of the impact of smoking, vaping and NRT on breastmilk in terms of composition and what enters milk | Nicotine & infant health | May include health effects of nicotine on infants, or the presence of nicotine in milk. |
| | | Reducing the risk | Mediating any perceived risks of breastfeeding a vaping by modifying vaping behaviour |
| | | Vaping & breastfeeding Vs Smoking & breastfeeding | Comparing the two behaviours either as a way of justifying the use of one or arguing the safety of one. |
| Risk to health | Discussing the concept of 'risk' involved in using ENDS as a new mother. Discussions of the safety of e-cigs and potential health implications of use. | A justified risk | Balancing up the perceived pro's & cons of vaping and breastfeeding, or justifying the use of an e-cig in a way to still adhere to 'good mother' roles. |
| | | Infant health | Discussions on possible positive or negative health implications of vaping as a breastfeeding mother on the infant. |
| | | Mothers health | Includes health effects for the mother- positive and negative, as well as discussions around addiction |
| | | Mediate the risk | Modifying behaviour or taking action to mediate any perceived risks that are non-specific to breastfeeding |
| Use | Exploring how women are using e-cigs and what the motivations are for use | Relapse prevention | E-cigs as a tool for reducing the risk of relapse to smoking |
| | | Quitting | As a tool to quit smoking |
| | | Motivation for use | Context-specific triggers for use and the concept of identity and choice |
| | | Alternatives | Alternatives to vaping offered |

Appendix 2 – Integrative themes - mapping

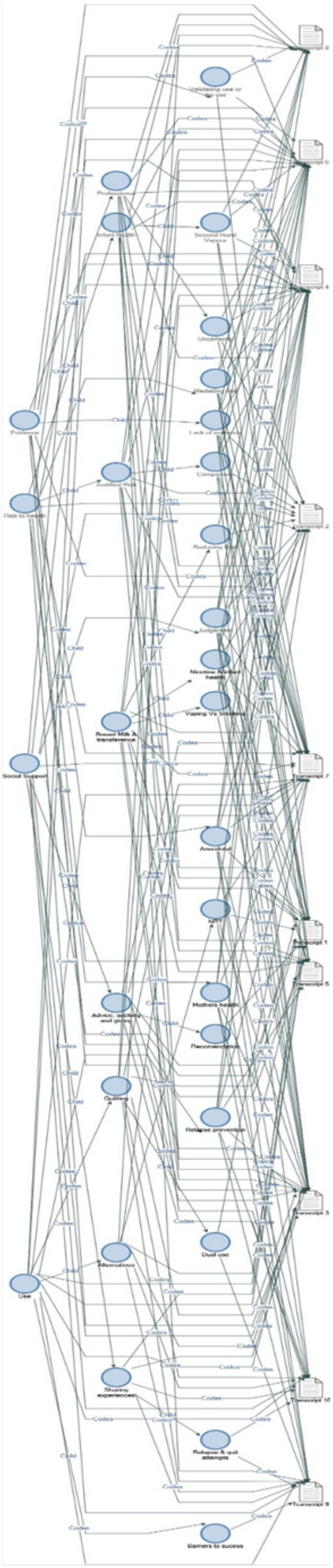

Supplement: Multimedia Appendix 1 [file jmir_v21i8e11506_app1.pdf]
