# Supplementary figures and images for: Safety of Electronic Cigarette Use During Breastfeeding: Qualitative Study Using Online Forum Discussions
Source: J Med Internet Res. 2019 Aug 12;21(8):e11506. doi: 10.2196/11506 (PMC6709563; doi:10.2196/11506)

## Appendix 2 – Integrative themes - mapping

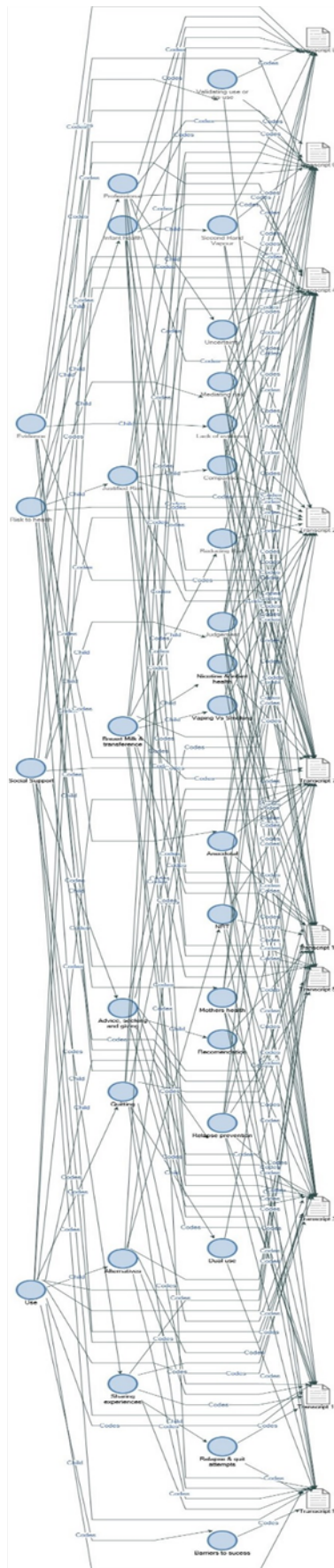

Supplement: Multimedia Appendix 2 [file jmir_v21i8e11506_app2.pdf]
